# Gestational body weight gain and risk of low birth weight or macrosomia in women of Japan: a nationwide cohort study

**DOI:** 10.1038/s41366-021-00947-7

**Published:** 2021-09-01

**Authors:** Hiroyuki Uchinuma, Kyoichiro Tsuchiya, Tetsuo Sekine, Sayaka Horiuchi, Megumi Kushima, Sanae Otawa, Hiroshi Yokomichi, Kunio Miyake, Yuka Akiyama, Tadao Ooka, Reiji Kojima, Ryoji Shinohara, Shuji Hirata, Zentaro Yamagata, Japan Environment and Children’s Study Group, Michihiro Kamijima, Shin Yamazaki, Yukihiro Ohya, Reiko Kishi, Nobuo Yaegashi, Koichi Hashimoto, Chisato Mori, Shuichi Ito, Zentaro Yamagata, Hidekuni Inadera, Takeo Nsakayama, Hiroyasu Iso, Masayuki Shima, Youichi Kurozawa, Narufumi Suganuma, Koichi Kusuhara, Takahiko Katoh

**Affiliations:** 1 Department of Diabetes and Endocrinology, University of Yamanashi Hospital, Yamanashi, Japan; 2 Center for Birth Cohort Studies, University of Yamanashi, Chuo City, Yamanashi, Japan; 3 Department of Health Sciences, School of Medicine, University of Yamanashi, Chuo City, Yamanashi, Japan; 4 Department of Obstetrics and Gynecology, University of Yamanashi, Chuo City, Yamanashi, Japan; 5 Nagoya City University, Nagoya, Japan; 6 National Institute for Environmental Studies, Tsukuba, Japan; 7 National Center for Child Health and Development, Tokyo, Japan; 8 Hokkaido University, Sapporo, Japan; 9 Tohoku University, Sendai, Japan; 10 Fukushima Medical University, Fukushima, Japan; 11 Chiba University, Chiba, Japan; 12 Yokohama City University, Yokohama, Japan; 13 University of Yamanashi, Chuo, Japan; 14 University of Toyama, Toyama, Japan; 15 Kyoto University, Kyoto, Japan; 16 Osaka University, Suita, Japan; 17 Hyogo College of Medicine, Nishinomiya, Japan; 18 Tottori University, Yonago, Japan; 19 Kochi University, Nankoku, Japan; 20 University of Occupational and Environmental Health, Kitakyushu, Japan; 21 Kumamoto University, Kumamoto, Japan

**Keywords:** Risk factors, Epidemiology

## Abstract

**Objective:**

Both maternal prepregnancy body mass index (BMI) and gestational weight gain (GWG) influence maternal and pediatric outcomes. We sought to clarify the impact of prepregnancy BMI-specific GWG and its patterns on the risk of low birth weight (LBW) or macrosomia using data from a large nationwide study in Japan.

**Methods:**

This cohort study (*n* = 98,052) used data from the Japan Environment and Children’s Study (JECS). The outcome variables in this study were LBW and macrosomia. We stratified the subjects into groups according to prepregnancy BMI.

**Results:**

GWG from pre-pregnancy to the first trimester had a small effect on the risk of LBW and macrosomia. From the first to second trimesters, insufficient GWG was associated with the risk of LBW, and from the second trimester to delivery, a GWG of less than 2 kg was associated with the risk of LBW. These associations were commonly observed in all prepregnancy BMI categories. Irrespective of the GWG from pre-pregnancy to the first trimester, GWG from the first to second trimesters affects LBW and/or macrosomia. Irrespective of the GWG from the first to second trimesters, GWG from the second trimester to delivery affects LBW and/or macrosomia. LBW or macrosomia was associated with the prevalence of a sustained low or high BMI percentile until three years of age, respectively.

**Conclusions:**

The present large national cohort study indicates that the risk of LBW or macrosomia is associated with GWG in women in Japan; the significance of this risk depends on the GWG patterns.

## Introduction

Both maternal prepregnancy body mass index (BMI) and gestational weight gain (GWG) influence maternal and pediatric outcomes. Several studies have reported that increasing prepregnancy body weight (BW) has a linear relationship with birth weight [1, 2]; consequently, an obese gravida is at an increased risk of delivering an infant with macrosomia. Conversely, women who are underweight when they conceive and have inadequate GWG are at an increased risk for delivering low birth weight (LBW) infants, which can have both short- and long-term consequences [3–5]. Although there is no global consensus on the recommended amount of GWG, the Institute of Medicine (IOM) guidelines are the most widely used in the world [6]. These guidelines offer specific weekly (kg/wk) and absolute (total kg) GWG gain recommendations based on a woman’s pregravid BMI [7]; they also provide specific ranges of weight gain for overweight and obese women, which were previously lacking [8]. However, it is difficult to compare, translate, or generalize the IOM weight gain recommendations to Asian women. Indeed, Japan has not adopted the IOM guidelines but has instead developed and adhered to original domestic guidelines, which are considerably stricter than the IOM guidelines. However, the guidelines have been questioned as to whether the strict GWG recommendations have contributed to the increasing rate of LBW infants in the country.

As well as total GWG, GWG across specific time intervals of pregnancy may contribute differently to perinatal outcomes. Weight gain in early pregnancy is reportedly a determinant of infant birth weight and is associated with maternal and neonatal complications [9, 10]; conversely, some studies have declared that weight gain during mid- and late pregnancy has a more important effect on infant birth weight since maternal GWG in these periods reflects fetal growth [11, 12]. In clinical settings, women who gain excessive weight before the second trimester often suppress weight gain after the second trimester. However, there has been no evidence whether the changes in GWG patterns affect the risk of LBW or macrosomia in Asian women with a large sample size.

In this context, we sought to clarify the impact of prepregnancy BMI-specific GWG and its patterns on the risk of LBW or macrosomia using a large nationwide study in Japan: the Japan Environment and Children’s Study (JECS), which is a nationwide birth cohort study founded by the Ministry of the Environment of Japan. The primary aim of the JECS is to analyze the effect of environmental risk factors on children’s health. This project is being conducted in 15 Regional Centers across Japan (Hokkaido, Miyagi, Fukushima, Chiba, Kanagawa, Koshin, Toyama, Aichi, Kyoto, Osaka, Hyogo, Tottori, Kochi, Fukuoka, and south Kyushu/Okinawa). Researchers recruited expectant mothers from these areas between 2011 and 2014, during which approximately 100,000 pregnancies were registered. Additionally, we tracked the infants’ BMI until three years of age to evaluate the relationship between mothers’ GWG and its patterns and their children’s growth.

## Materials and methods

### Study sample

Data from the JECS, a government-funded birth cohort study started in January 2011, were used. This survey investigated the effect of several environmental factors on children’s health [13]. The eligibility requirements of JECS participants (mothers) were as follows: 1) living in the Study Area at the time of application and were expected to live in Japan in the near future; 2) expected delivery date between 1 August 2011 and mid-2014; and 3) could participate without difficulty (i.e., they could answer the self-management questionnaire). In this study, we used the jecs-ta-20190930 dataset released in October 2019. From a total of 104,062 fetal records, we excluded 1,636 cases of miscarriage or stillbirth, 1,891 cases of multiple births, and 2,483 cases with missing data regarding birthweight. Finally, the data for 98,052 participants were analyzed in this study (Supplementary Fig. 1).

The authors assert that all procedures contributing to this work comply with the ethical standards of the relevant national and institutional committees on human experimentation and with the Declaration of Helsinki of 1975, as revised in 2008. The JECS protocol was reviewed and approved by the Ministry of the Environment’s Institutional Review Board on Epidemiological Studies and the Ethics Committees of all participating institutions, and the Ethics Committee, University of Yamanashi. Written informed consent was obtained from all participants.

### Measures

The outcome variables in this study were LBW, macrosomia, and moderate and severe hypertensive disorders in pregnancy (HDP). Small for gestational age was defined as a weight below the 10th percentile for the gestational age. LBW has been defined as the first weight recorded within hours of birth of <2500 g. Very LBW is defined as <1500 g and extremely LBW is defined as <1000 g. Macrosomia was defined as ≥4000 g. All data on maternal BW during pregnancy were based on BW at prenatal checkups during the JECS. prepregnancy BMI was self-reported in the questionnaire responses. Maternal BW measured at gestational age 0w0d to 13w6d was considered “BW at first trimester”. All maternal BW measurements from gestational age 14w0d to 27w6d were considered “BW at second trimester”. All maternal BW measurements from gestational age 28w0d until delivery were considered “BW at third trimester”. Weight gain during the total gestational period (total GWG) and specific intervals are defined in Supplementary Fig. 2.

Information on HDP, which was defined as systolic blood pressure ≥140 mmHg and/or diastolic blood pressure ≥ 90 mmHg in this study, was obtained from the Dr0m questionnaire. Since this questionnaire only revealed whether the participant was diagnosed with HDP or not, we analyzed the incidence of HDP without a previous history of hypertension before pregnancy. We also defined severe HDP as systolic blood pressure ≥ 160 mmHg, diastolic blood pressure ≥ 110 mmHg, and/or proteinuria (≥ 2 g/day).

### Statistical analysis

We calculated prepregnancy BMI from self-reported prepregnancy height and BW, and we stratified the subjects into five groups based on BMI: <18.5, ≥18.5 to <21, ≥21 to <25, ≥25 to <30, and ≥30 kg/m^2^. In some analyses, we stratified the subjects into four groups because of small numbers of outcome events.

First, we performed univariate and multivariate logistic regression analyses on LBW or macrosomia with known basic confounders: when LBW was used as a dependent variable, the independent variables were age, prepregnancy height, and BMI, total GWG, smoking status, gestational week at delivery, birth history, and HDP. When macrosomia was used as a dependent variable, the independent variables were prepregnancy height and BMI, total GWG, gestational week of delivery, and history of macrosomia and diabetes. When HDP was used as a dependent variable, the independent variables were age, prepregnancy height and BMI, total GWG, gestational weeks of delivery, and history of LBW, HDP, diabetes, hypertension, hyperthyroidism, hypothyroidism, systemic lupus erythematosus (SLE), and antiphospholipid antibody syndrome.

Next, the associations between the risk of LBW or macrosomia and maternal BW gain were evaluated using a multinomial logistic regression analysis with LBW or macrosomia as the dependent variables. We categorized the independent variable maternal BW gain into six groups during specific time intervals.

Furthermore, to assess the associations between the risk of LBW and macrosomia and GWG patterns, we performed a multinomial logistic regression analysis using LBW, macrosomia, or HDP as dependent variables. We used BW gain from prepregnancy to the first trimester and from the first to second trimesters as categorical independent variables.

We finally tracked the BMI of children born from the subjects in this study until three years of age. The associations between LBW or macrosomia and the risk of a BMI below the 10th percentile or above the 90th percentile at 1, 1.5, 2, 2.5, and 3 years old were evaluated using a multinomial logistic regression analysis with LBW or macrosomia as dependent variables. The independent variables included the duration of breastfeeding and a parent’s total number of cigarettes smoked per day in the home. Statistical adjustment was performed by conventional confounding factors [14–16]. Growth standards for Japanese children with percentile values based on the year 2000 national survey data, which have been recommended as reference data in Japan, were used as the standard.

All descriptive and statistical analyses were performed using Microsoft Excel 2016 (Microsoft, Redmond, WA, USA) and Easy R (EZR; Saitama Medical Center, Jichi Medical University, Saitama, Japan). Statistical significance was set at 0.01 or 0.0001, and all statistical tests were two-tailed.

## Results

### Subject characteristics

A total of 98,052 subjects were classified into the following five categories according to their prepregnancy BMI: 16.2% (*n* = 15,845) had a BMI < 18.5 kg/m^2^, 41.1% (*n* = 40,294) had a BMI ≥ 18.5 to <21 kg/m^2^, 31.9% (*n* = 31,275) had a BMI ≥ 21 to <25 kg/m^2^, 8.2% (*n* = 8035) had a BMI ≥ 25 to <30 kg/m^2^, and 2.5% (*n* = 2,477) had a BMI ≥ 30 kg/m^2^ (Supplementary Fig. 1). Whereas the proportion of LBW infants was significantly higher in the lower BMI groups, that of infants with macrosomia was significantly higher in the higher BMI groups (Table 1).

**Table 1:** Characteristics of participants enrolled in the Japan Environment and Children’s Study.

			Prepregnancy BMI (kg/m²)					*p* for trend
	Units	All	<18.5	18.5 to <21	21 to <25	25 to <30	≥30	
	*n* (%)	*n* = 98,052	*n* = 15,845 (16.2)	*n* = 40,294 (41.1)	*n* = 31,275 (31.9)	*n* = 8035 (8.2)	*n* = 2477 (2.5)	
Infants								
Sex	*n* (%)							
Male		50,242 (51.2)	8177 (51.6)	20,594 (51.1)	15,968 (49.6)	4186 (52.1)	1259 (50.8)	
Female		47,803 (48.8)	7668 (48.4)	19,700 (48.9)	16,207 (50.4)	3849 (47.9)	1218 (49.2)	*
Birthweight	g	3023.2 ± 419.6	2922.0 ± 399.3	3007.7 ± 397.1	3063.6 ± 423.6	3106.5 ± 465.2	3142.7 ± 517.6	
LBW	*n* (%)	7967 (8.1)	1826 (11.5)	3155 (7.8)	2193 (7.0)	582 (7.2)	191 (7.7)	**
VLBW	*n* (%)	575 (0.6)	98 (0.6)	189 (0.5)	175 (0.6)	73 (0.9)	34 (1.4)	**
ELBW	*n* (%)	243 (0.2)	33 (0.2)	82 (0.2)	79 (0.3)	28 (0.3)	18 (0.7)	**
SGA	*n* (%)	7359 (7.7)	1746 (11.3)	3094 (7.9)	1951 (6.4)	456 (5.8)	111 (4.6)	**
Macrosomia	*n* (%)	858 (0.9)	44 (0.3)	222 (0.6)	355 (1.1)	160 (2.0)	75 (3.0)	**
Gestational age	weeks	38.8 ± 1.6	38.7 ± 1.6	38.9 ± 1.5	38.9 ± 1.6	38.8 ± 1.8	38.6 ± 2.0	
<37	*n* (%)	4604 (4.7)	842 (5.3)	1682 (4.2)	1410 (4.5)	462 (5.7)	194 (7.8)	**
37 to <42	*n* (%)	93,221 (95.1)	14,978 (94.5)	38,543 (95.7)	29,768 (95.2)	7551 (94.0)	2272 (91.7)	**
≥42	*n* (%)	225 (0.2)	25 (0.2)	69 (0.2)	97 (0.3)	22 (0.3)	11 (0.4)	**
Mothers								
Age	years	30.7 ± 5.1	29.8 ± 5.0	30.7 ± 5.1	31.1 ± 5.1	31.3 ± 5.2	31.4 ± 5.0	
<20	*n* (%)	1128 (1.2)	268 (1.7)	461 (1.2)	313 (1.0)	75 (1.0)	10 (0.4)	**
20 to <25	*n* (%)	10,006 (10.4)	2119 (13.6)	4103 (10.4)	2,846 (9.3)	728 (9.3)	205 (8.6)	**
25 to <30	*n* (%)	28,357 (29.5)	5077 (32.6)	11,963 (30.2)	8581 (28.0)	2081 (26.6)	635 (26.6)	**
30 to <35	*n* (%)	33,470 (34.8)	5169 (33.2)	14,052 (35.5)	10,638 (34.7)	2737 (34.9)	853 (35.7)	
35 to <40	*n* (%)	19,739 (20.5)	2569 (16.5)	7792 (19.7)	6953 (22.7)	1830 (23.4)	581 (24.3)	**
≥40	*n* (%)	3360 (3.5)	358 (2.3)	1190 (3.0)	1322 (4.3)	384 (4.9)	104 (4.4)	**
Height	cm	158.1 ± 5.4	158.5 ± 5.4	158.3 ± 5.3	157.8 ± 5.4	157.6 ± 5.5	158.1 ± 5.5	
Parity	*n* (%)							
Primiparous		38,499 (40.2)	6736 (43.7)	16,383 (41.7)	11,855 (38.8)	2680 (34.0)	818 (33.6)	**
Multiparous		57,184 (59.8)	8685 (56.3)	22,908 (58.3)	18,725 (61.2)	5207 (66.0)	1620 (66.4)	**
Smoking status	*n* (%)							
Never smoker		55,224 (57.8)	9083 (58.8)	23,595 (59.9)	17,341 (52.3)	4077 (50.7)	1101 (46.1)	**
Ex-smoker, stopped before learning of pregnancy		22,872 (23.9)	3239 (21.0)	9190 (23.3)	7752 (25.9)	2016 (25.1)	658 (27.5)	**
Ex-smoker, stopped on awareness of pregnancy		13,097 (13.7)	2310 (15.0)	5069 (12.9)	4059 (15.4)	1204 (15.0)	442 (18.5)	*
Current smoker		4408 (4.6)	814 (5.3)	1524 (3.9)	1377 (6.4)	500 (6.2)	189 (7.9)	**
Total GWG	kg	10.3 ± 4.9	10.9 ± 5.4	10.7 ± 3.6	10.3 ± 4.1	8.5 ± 9.0	5.2 ± 5.7	
<7	*n* (%)	16,392 (17.1)	1679 (10.8)	4786 (12.1)	5455 (17.8)	2948 (37.2)	1516 (62.1)	**
7 to <9	*n* (%)	16,855 (17.6)	2685 (17.3)	7016 (17.8)	5446 (17.7)	1363 (17.2)	337 (13.8)	
9 to <12	*n* (%)	31,894 (33.2)	5591 (36.1)	14,181 (36.0)	9880 (32.2)	1877 (23.7)	351 (14.4)	**
≥12	*n* (%)	30,843 (32.1)	5527 (35.7)	13,412 (34.0)	9911 (32.3)	1734 (21.9)	237 (9.7)	**
Diabetes mellitus	*n* (%)	3411 (3.5)	329 (2.1)	910 (2.3)	1068 (3.4)	637 (7.9)	460 (18.6)	**
HDP	*n* (%)							
Moderate		2230 (2.3)	207 (1.3)	585 (1.5)	783 (2.5)	397 (4.9)	256 (10.3)	**
Severe		935 (1.0)	88 (0.6)	276 (0.7)	317 (1.0)	148 (1.8)	104 (4.2)	**
History of delivery of macrosomia	*n* (%)	621 (0.6)	41 (0.3)	177 (0.5)	237 (0.8)	114 (1.4)	52 (2.1)	**

*BMI* body mass index, *LBW* low birth weight infant, *ELBW* extremely low birth weight infant, *VLBW* very low birth weight infant, *SGA* small for gestational age *GWG*, gestational weight gain, *HDP* hypertensive disorders of pregnancy.

*p < 0.01

**p < 0.0001.

### Univariate and multivariate analyses on known risk factors for LBW and macrosomia

The univariate and multivariate analyses on known risk factors for LBW (Supplementary Table 1) and macrosomia (Supplementary Table 2) were consistent with those of previous studies [17–22]. Furthermore, late gestational age, high prepregnancy BMI, and large total GWG were associated with the risk of macrosomia. Similarly, the results of the univariate and multivariate analyses on known risk factors for light-for-date infants were similar to those for LBW infants (Supplementary Table 1). The univariate and multivariate analyses on known risk factors for moderate and severe hypertensive disorders of pregnancy (HDP) almost corroborated the findings of previous studies [23, 24]; however, some diseases in their medical history (hyperthyroidism, SLE, and antiphospholipid syndrome), which were previously reported as risk factors for HDP [25–29], were not revealed as significant risk factors in the subjects of the present study (Supplementary Table 3).

*Risk of LBW and macrosomia according to GWG from pre-pregnancy to each term of pregnancy*.

**Fig. 1:**
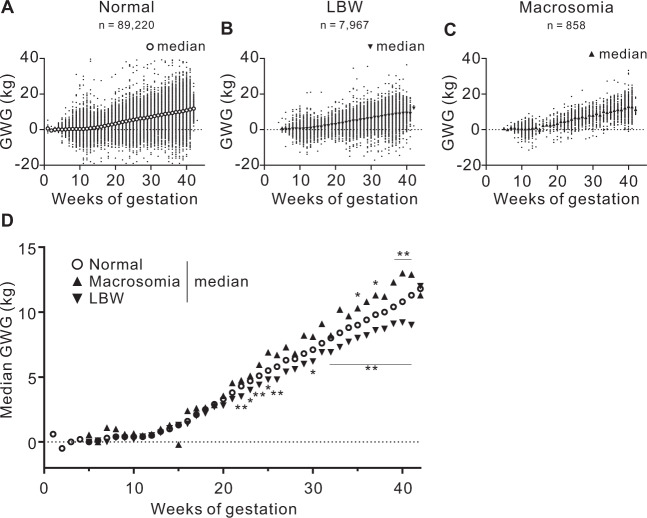
GWG in subjects who delivered infants with LBW, normal weight, and macrosomia. Relationships between GWG and weeks of gestation in subjects who delivered infants with **A** normal weight (*n* = 89,220), **B** LBW (*n* = 7967), and **C** macrosomia (*n* = 858). Large symbols indicate the median BW gain for the week of gestation since pre-pregnancy. **D** Median BW gain of each week of gestation of the subjects who delivered infants with LBW, normal weight, and macrosomia with fitted nonlinear regression curves. Figure 1A–C was merged of GWG of all subjects recorded a time in each trimester. GWG, gestational weight gain; LBW, low birth weight. *p < 0.01 and **p < 0.0001 vs. normal by ANOVA with post-hoc Dunnett’s test.

**Fig. 2:**
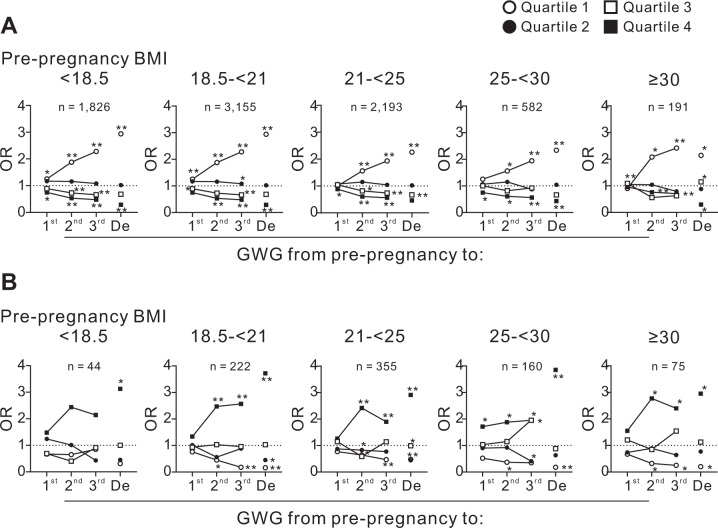
The odds ratios for LBW and macrosomia according to GWG from pre-pregnancy to the first, second, and third trimesters and delivery. The odds ratios for **A** LBW and **B** macrosomia in the five prepregnancy BMI categories according to GWG from pre-pregnancy to the first, second, and third trimesters and delivery are shown. The GWG from pre-pregnancy to the first (1st), second (2nd), and third (3rd) trimesters and delivery (De) was categorized into quartiles (Quartile 1, 2, 3, and 4). GWG, gestational weight gain; OR, odds ratio. *p < 0.01 and **p < 0.0001.

Figure 1 displays a scatter plot for GWG at each week of pregnancy in subjects who delivered LBW, normal-weight infants, or infants with macrosomia. After 22 weeks, GWG was greater in the subjects who delivered macrosomia infants and lower in the subjects who delivered LBW infants. The GWG from pre-pregnancy to the first, second, and third trimesters and to delivery were categorized into quartiles (Quartiles 1, 2, 3, and 4); subsequently, a multiple logistic regression analysis was performed to assess the risk of LBW or macrosomia associated with GWG from pre-pregnancy (Fig. 2A and B). The odds ratios (ORs) of the risks of LBW (Fig. 2A) and macrosomia (Fig. 2B) for GWG from pre-pregnancy to the first trimester were relatively low in all four prepregnancy BMI groups. However, after the second trimester, the ORs for LBW and macrosomia increased in the Quartile 1 group, which indicated the smallest GWG during the period (Fig. 2A and B).

### Risk of LBW and macrosomia according to GWG during specific intervals

**Fig. 3:**
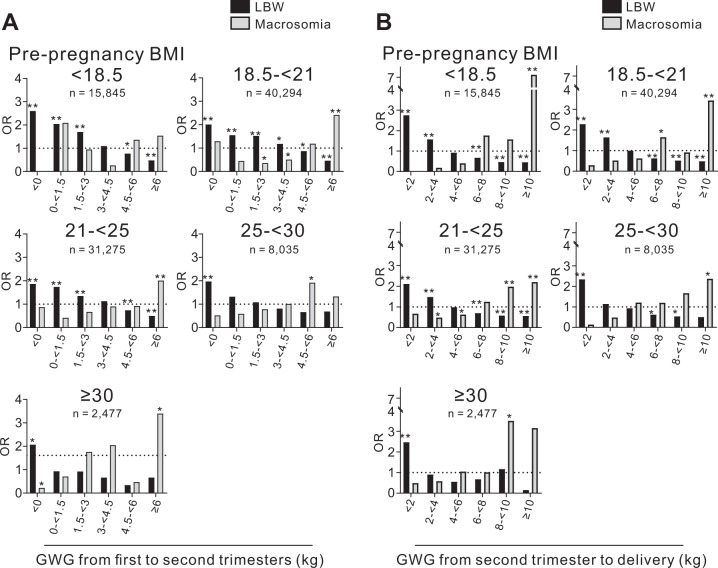
The odds ratios for LBW and macrosomia according to GWG during specific intervals of pregnancy. The odds ratios for LBW and macrosomia in the five prepregnancy BMI categories according to GWG **A** from the first to second trimester and **B** from the second trimester to delivery are shown. GWG, gestational weight gain; LBW, low birth weight; OR, odds ratio. *p < 0.01 and **p < 0.0001.

We next assessed the risk of LBW or macrosomia in association with GWG during the following three specific intervals: from pre-pregnancy to the first trimester, from the first to second trimesters, and from the second trimester to delivery. GWG from pre-pregnancy to the first trimester had a small effect on the proportion of LBW and macrosomia infants in all prepregnancy BMI groups (Supplementary Fig. 3). However, GWG from the first to second trimesters (Fig. 3A) and from the second trimester to delivery (Fig. 3B) was negatively associated with the risk of LBW and positively associated with the risk of macrosomia. Importantly, there were ranges of GWG that did not significantly increase the risk of LBW or macrosomia.

### Risk of LBW and macrosomia according to GWG patterns

**Fig. 4:**
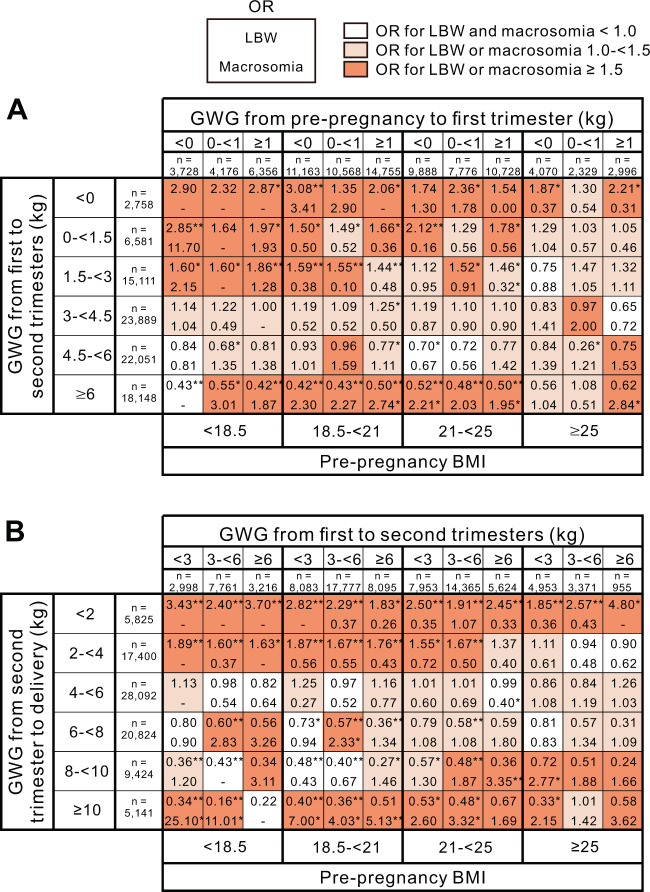
The odds ratio of LBW and macrosomia according to GWG patterns. **A** The upper horizontal axis shows three categories according to GWG from pre-pregnancy to the first trimester. The bottom horizontal axis shows four categories according to the prepregnancy BMI. The vertical axis shows six categories according to GWG from the first to second trimesters. **B** The upper horizontal axis shows three categories according to GWG from the first to second trimesters. The bottom horizontal axis shows four categories according to the prepregnancy BMI. The vertical axis shows six categories according to GWG from the second trimester to delivery. The odds ratios of LBW (upper rows in cells) and macrosomia (lower rows in cells) in the 3 ×6 patterns of GWG for each prepregnancy BMI group are shown. The cells are colored to indicate increases in the OR for LBW and/or macrosomia. GWG, gestational weight gain; BMI, body mass index; LBW, low birth weight; OR, odds ratio. *p < 0.01 and **p < 0.0001.

In the women with a pre-BMI < 25 kg/m^2^, subjects whose GWG was <3 kg from the first to second trimesters tended to gain more BW from the second trimester to delivery than in those whose GWG was ≥3 kg from the first to second trimesters (Supplementary Fig. 4). Conversely, in subjects with a prepregnancy BMI ≥ 25 kg/m^2^, the proportion of subjects whose GWG was <2 kg from the second trimester to delivery was higher than that in other categories. Therefore, in this study, subjects may intentionally control GWG after the second trimester based on GWG before the second trimester to approach the target total GWG. Then, we assessed whether the patterns of GWG affected the risk of LBW and macrosomia from four prepregnancy BMI categories, the subjects were classified into three categories according to their GWG from pre-pregnancy to the first trimester. Then, the subjects were further classified into six categories according to their GWG from the first to second trimesters (Fig. 4A). We consequently assessed the risk of LBW and macrosomia in the 3 ×6 patterns of GWG using a multiple logistic regression analysis. In the groups with a prepregnancy BMI < 25 kg/m^2^, no or relatively small increases in the risk for LBW and macrosomia were observed when the GWG from the first to second trimesters was ≥3 to <6 kg, irrespective of the category of GWG from pre-pregnancy to the first trimester (Fig. 4A). In the group with a BMI ≥ 25 kg/m^2^, no or relatively small increases in the risks of LBW and macrosomia were observed when the GWG from the first to second trimesters was ≥0 to <3 kg, independent of the category of GWG from pre-pregnancy to the first trimester.

Similarly, in the women with a prepregnancy BMI < 25 kg/m^2^, no or relatively small increases in the risks of LBW and macrosomia were observed when the GWG from the second trimesters to delivery was ≥4 to <6 kg, irrespective of the category of GWG from the first to second trimesters (Fig. 4B). In the women with a prepregnancy BMI ≥ 25 kg/m^2^, no or relatively small increases in the risks of LBW and macrosomia were observed when the GWG from the second trimesters to delivery was ≥2 to <8 kg, independent of the category of GWG from the first to second trimesters. In addition, in the women with a prepregnancy BMI ≥ 25 kg/m^2^, no or relatively small increases in the risks of LBW and macrosomia were observed when the GWG from the second trimester to delivery was ≥2 to <8 kg, independent of the category of GWG from the first to second trimesters.

### Risk of HDP according to GWG patterns

To clarify whether the optimal patterns of maternal BW gain during the gestational period to avoid LBW and macrosomia affects the risk of other maternal adverse events, we assessed the risk of moderate and severe HDP as an example of an adverse maternal event reportedly associated with GWG [30, 31]. In all pre-BMI categories, a significant increase in the risk for HDP was hardly observed in any categories of GWG from pre-pregnancy to the first trimester and from the first to second trimesters (Supplementary Fig. 5). However, a greater GWG from the second trimester to delivery was associated with an increased risk for HDP. Furthermore, the risks of HDP according to the GWG from second trimester to delivery were observed in any category of GWG from first to second trimesters (Supplementary Fig. 6). Notably, no significant risk elevation for HDP was observed in GWG from the second trimester to delivery in the GWG ranges that did not significantly increase the risk for both LBW and macrosomia (≥4 to <8 kg in the BMI < 18.5 kg/m^2^ group, ≥4 to <6 kg in the ≥18.5 to <25 kg/m^2^ group, and ≥2 to <8 kg in the ≥ 25 kg/m^2^ group).

### Three-year follow-up of BMI in children born at LBW or macrosomia

**Fig. 5:**
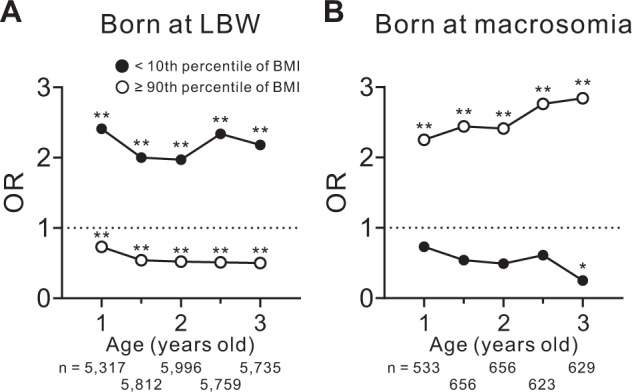
The odds ratio of low or high BMI according to the age of the children born at LBW or macrosomia. The odds ratio for a BMI below the 10^th^ percentile (closed circles) or above the 90th percentile (open circles) at the indicated age in children born at **A** LBW (*n* = 7,967) or **B** macrosomia (*n* = 858). BMI, body mass index; LBW, low birth weight; OR, odds ratio. *p < 0.01 and **p < 0.0001.

Finally, we analyzed the risk for low (<10th percentile) or high (≥90th percentile) BMI until three years of age in the children born at LBW or macrosomia. Children born at LBW consistently showed a low risk for high BMI values at 1, 1.5, 2, 2.5, and 3 years old, but they had a high risk for low BMI values (Fig. 5A). Conversely, children born at macrosomia consistently showed a high risk for high BMI values until three years of age but had a low risk for low BMI values at three years (Fig. 5B).

## Discussion

As observed in western countries including the United States [32], France [33], and Germany [34], the average birth weight in Japan has been decreasing every year. The prevalence of LBW has increased from 5.2% in 1980 to 9.6% in 2005, representing a 1.8-fold increase in 25 years [35, 36]. By contrast, although its prevalence in Japan (0.8%) is much lower than the values in US neonates (7.6–13%) [37, 38], macrosomia is also associated with an increased risk of for long-term complications, such as obesity and insulin resistance [39]. Maternal obesity and excessive GWG now appear to have a greater impact on the prevalence of macrosomia than maternal diabetes [17, 21]. Thus, to avoid increasing the risk of both LBW and macrosomia, race- and region-specific indicators for GWG based on large cohort studies are desired. Using data of the JECS cohort, Jung et al. have reported that environmental toxicants such as heavy metals modify the associations between total GWG and pregnancy outcomes including LBW, macrosomia, and HDP [40]. Their observations related to the optimal GWG for pregnancy outcomes are consistent with our observation. However, the impact of prepregnancy BMI-specific GWG patterns on the risk of LBW or macrosomia had remained unclear.

The following findings were commonly observed in the present study in all prepregnancy BMI categories: (1) from pre-pregnancy to the first trimester, GWG had a small effect on the risk of LBW or macrosomia, (2) from the first to second trimesters, insufficient GWG was associated with the risk of LBW, and (3) from the second trimester to delivery, a GWG of less than 2 kg was associated with the risk of LBW. Based on our observations, even in subjects with a prepregnancy BMI ≥ 25 kg/m^2^, which is diagnosed as “obese” by Japanese criteria, insufficient GWG after the second trimester can increase the risk of LBW.

This study also showed that the GWG pattern significantly affected the risk of LBW and/or macrosomia in women of Japan. Irrespective to GWG from pre-pregnancy to the first trimester, GWG from the first to second trimesters affects LBW and/or macrosomia. Furthermore, irrespective of the GWG from the first to second trimesters, GWG from the second trimester to delivery affects LBW and/or macrosomia. These observations suggest that to minimize the risk of LBW and macrosomia, the appropriate GWG must be determined during multiple intervals of the gestation period. The Japanese Ministry of Health, Labour and Welfare guideline provide two main recommendations for GWG, which are the total GWG and the weekly GWG after 14w0d. However, no specific total GWG and weekly GWG after 14w0d are recommended for subjects with a prepregnancy BMI ≥ 25 kg/m^2^, which accounted for 10.7% of the total subjects in this study. The IOM guidelines report recommendations for the total GWG and weekly GWG in the second and third trimesters for overweight (BMI ≥ 25 to <30 kg/m^2^) and obese (BMI ≥ 30 kg/m^2^) subjects [6]. However, independent of GWG, Asian women reportedly have a higher predicted probability for LBW compared with white women and a lower predicted probability for macrosomia compared with white women [41]. Indeed, based on our analysis, if subjects with a BMI ≥ 25 to <30 kg/m^2^ in this study gained 14.1–22.7 kg, which is the recommended total GWG for overweight women, it will result in an increased risk of macrosomia.

A possible mechanism by which GWG patterns are associated with the risk of LBW or macrosomia is epigenetic modifications in nonimprinted genes induced by aspects of the developmental environment [42]; during the 1944–1945 famine in the Netherlands, the periconceptional exposure to famine was associated with lower methylation of the differentially methylated regions (DMRs) in insulin-like growth factor 2 (IGF2) gene. In contrast, exposure during the late gestational phase was not associated with methylation in IGF2 DMRs [43]. Epigenetic marks may be particularly vulnerable during the very early stage of mammalian development that represents a crucial period for establishing and maintaining epigenetic marks [44].

In this study, the subjects seem to focus on the total GWG more than the GWG patterns, which may result in insufficient or excessive GWG after the second trimester. However, our observation suggests that in all prepregnancy BMI categories, GWG after the second trimester affects the risk of LBW and/or macrosomia, irrespective of GWG until the second trimester. Thus, if women focus too much on the total GWG, there may be an increased risk of LBW and/or macrosomia for women in Japan.

The 3-year follow-up of the children’s BMI suggested that being born at LBW or macrosomia increases the risk of being in the low or high BMI percentiles until 3 years of age, respectively. Importantly, small size at birth per se is well known to be associated with early adiposity rebound, obesity, and metabolic syndrome [45–49]. Although, the present study was able to analyze the children’s BMI until 3 years of age, further observations of the children’s cohort may show the prevalence of early adiposity rebound in LBW infants and the relationship between LBW and the future prevalence of obesity and its comorbidities.

In addition to LBW and macrosomia, we assessed the risk of HDP as an adverse event related to both prepregnancy BMI and GWG. Maternal BMI is consistently reported as an independent risk factor for both preeclampsia and gestational hypertension [50–52]. The present study showed that GWG that did not increase the risk of both LBW and macrosomia was not associated with the risk of HDP in women in Japan. Thus, it suggests that the optimal GWG for avoiding the risk of both LBW and macrosomia can coexist with the optimal GWG for avoiding the risk of HDP in women of Japan.

This study has some limitations. First, the BW at each trimester was defined as the BW measured at some point within the respective trimester at the JECS checkups. Since the specific timepoint in each trimester was not indicated, the intervals among pre-pregnancy, the first, second, and third trimesters, and delivery varied among individuals (Supplementary Fig. 2). Second, the number of macrosomia infants (*n* = 858) was smaller than the number of LBW (*n* = 7967) and adequate to gestational age (*n* = 89,220) infants. Therefore, the low number of cases limited the data analysis and ability to draw conclusions. Third, since the present study was a prospective cohort study, it does not demonstrate the effect of an intervention for maternal BW on the outcomes of infants. Fourth, prepregnancy height and BW, smoking history, past history of diseases, and children’s height and BW were self-reported values.

In conclusion, the present large national cohort study indicates that the risk of LBW or macrosomia is associated with GWG in women in Japan; the significance of this risk depends on the terms of pregnancy and the GWG pattern. The present study also implies that subjects may try to accelerate GWG after the second trimester based on the GWG before the second trimester to attempt to achieve the target total GWG, which consequently affects the risk of LBW and/or macrosomia. In any prepregnancy BMI category including BMI ≥ 25 kg/m^2^, which is diagnosed as obese by the Japanese criteria, insufficient GWG from the second trimester to delivery consistently increases the risk of LBW. The results of this study may serve as a standard for the optimal GWG for women in Japan and possibly for Asian women in general.

## Supplementary information

Supplementary Information
