# Supplementary material for: Gestational body weight gain and risk of low birth weight or macrosomia in women of Japan: a nationwide cohort study
Source: Int J Obes (Lond). 2021 Sep 1;45(12):2666–74. doi: 10.1038/s41366-021-00947-7 (PMC8606312; doi:10.1038/s41366-021-00947-7)
Supplement: Supplementary file 1 — Supplementary Information [file 41366_2021_947_MOESM1_ESM.pdf]

SUPPLEMENTARY INFORMATION

Maternal body weight gain and risk of low birth weight or macrosomia in women of Japan: a nationwide cohort study

Hiroyuki Uchinuma, Kyoichiro Tsuchiya, Tetsuo Sekine, Sanae Otawa, Ryoji Shinohara, Shuji Hirata, Zentaro Yamagata, and Japan Environment and Children's Study Group

This file includes Acknowledgement, Supplementary Tables 1-3, and Supplementary Figures 1-6.

ACKNOWLEDGEMENT

Appendix

Members of the JECS Group as of 2020: Michihiro Kamijima (principal investigator, Nagoya City University, Nagoya, Japan), Shin Yamazaki (National Institute for Environmental Studies, Tsukuba, Japan), Yukihiro Ohya (National Center for Child Health and Development, Tokyo, Japan), Reiko Kishi (Hokkaido University, Sapporo, Japan), Nobuo Yaegashi (Tohoku University, Sendai, Japan), Koichi Hashimoto (Fukushima Medical University, Fukushima, Japan), Chisato Mori (Chiba University, Chiba, Japan), Shuichi Ito (Yokohama City University, Yokohama, Japan), Zentaro Yamagata (University of Yamanashi, Chuo, Japan), Hidekuni Inadera (University of Toyama, Toyama, Japan), Takeo Nakayama (Kyoto University, Kyoto, Japan), Hiroyasu Iso (Osaka University, Suita, Japan), Masayuki Shima (Hyogo College of Medicine, Nishinomiya, Japan), Youichi Kurozawa (Tottori University, Yonago, Japan), Narufumi Suganuma (Kochi University, Nankoku, Japan), Koichi Kusuhara (University of Occupational and Environmental Health, Kitakyushu, Japan), and Takahiko Katoh (Kumamoto University, Kumamoto, Japan).

Funding

This work was funded by the Ministry of the Environment, Japan. The findings and conclusions of this article are solely the responsibility of the authors and do not represent the official views of the Ministry of the Environment.

Disclosure Summary

The authors have declared that no competing interests exist.

Author Contributions

H.U., T.S., and K.T. designed the study, acquired and analyzed the data, and wrote the manuscript. Y.H., M.K., H.Y., K.M., S.O., Y.A., T.O., R.K., R.S., S.H., Z.Y., and the Japan Environment and Children's Study (JECS) Group designed the study, acquired data, and supervised the analysis. The JECS Group is the guarantor of this work and, as such, had full access to all the data in the study and takes responsibility for the integrity of the data and the accuracy of the data analysis.

Data Availability

Data are unsuitable for public deposition due to ethical restrictions and the legal framework of Japan. It is prohibited by the Act on the Protection of Personal Information (Act No.57 of 30 May 2003, amendment on September 9, 2015) to publicly deposit data containing personal information. The Ethical Guidelines for Epidemiological Research

enforced by the Japan Ministry of Education, Culture, Sports, Science and Technology and the Ministry of Health, Labour and Welfare also restrict the open sharing of the epidemiologic data. All inquiries about access to data should be sent to: jecs-en@nies.go.jp. The person responsible for handling enquiries sent to this e-mail address is Dr. Shoji F. Nakayama, JECS Programme Office, National Institute for Environmental Studies.

SUPPLEMENTARY TABLES

Supplementary Table 1. Unadjusted and multivariate adjusted predictors of LBW

Characteristic	LBW			
	Univariate		Multivariate	
	Unadjusted OR (95% CI)	*p*-value	Adjusted OR (95% CI)	*p*-value
Gestational age (weeks)				
<37	29.10 (27.20–31.00)	0.00	22.50 (20.80–24.20)	0.00
37 to <42	Ref		Ref	
≥42	0.23 (0.08–0.73)	1.25E-02	0.32 (0.10–0.99)	4.87E-02
Age (years)				
<20	1.20 (0.98–1.48)	8.15E-02	0.93 (0.72–1.20)	5.51E-01
20 to <25	0.96 (0.88–1.05)	3.35E-01	0.87 (0.78–0.96)	7.64E-03
25 to <30	Ref		Ref	
30 to <35	1.06 (1.00–1.12)	6.20E-02	1.07 (1.00–1.15)	4.84E-02
35 to <40	1.21 (1.13–1.29)	1.33E-08	1.09 (1.00–1.18)	3.88E-02
≥40	1.61 (1.43–1.80)	4.03E-16	1.38 (1.19–1.59)	1.10E-05
Height	0.95 (0.95–0.96)	2.33E-108	0.95 (0.94–0.95)	3.84E-88
Pre-pregnancy BMI (kg/m^2)				
<18.5	1.53 (1.44–1.63)	3.68E-43	1.61 (1.50–1.73)	2.07E-37
18.5 to <21	Ref		Ref	
21 to <25	0.89 (0.84–0.94)	3.69E-05	0.72 (0.67–0.77)	1.71E-21
25 to <30	0.92 (0.84–1.01)	7.23E-02	0.48 (0.43–0.54)	6.01E-36
≥30	0.98 (0.85–1.15)	8.30E-01	0.27 (0.22–0.33)	3.33E-36
Parity				
Primiparous	1.32 (1.26–1.38)	3.59E-31	1.53 (1.44–1.62)	1.21E-46
Multiparous	Ref		Ref	

Smoking status				
Never smoker	Ref		Ref	
Ex-smoker, stopped before learning of pregnancy	0.93 (0.88–0.98)	1.25E-02	1.03 (0.96–1.10)	4.63E-01
Ex-smoker, stopped on awareness of pregnancy	1.01 (0.94–1.08)	8.56E-01	1.36 (1.25–1.49)	1.43E-12
Current smoker	1.82 (1.66–2.00)	1.66E-37	2.53 (2.27–2.82)	3.29E-61
Total GWG (kg)				
<7	2.68 (2.52–2.85)	4.08E-223	2.52 (2.34–2.71)	2.36E-131
7 to <9	1.63 (1.52–1.74)	3.11E-47	1.53 (1.42–1.65)	2.25E-27
9 to <12	Ref		Ref	
≥12	0.56 (0.52–0.60)	3.60E-55	0.54 (0.49–0.58)	1.60E-48
HDP				
Moderate	3.06 (2.75–3.42)	9.77E-89	3.38 (2.94–3.89)	3.01E-66
Severe	10.70 (9.43–12.30)	1.60E-277	6.25 (5.19–7.52)	2.55E-83

LBW, low birth weight infant; OR, odds ratio; CI, confidence interval; BMI, body mass index; HDP, hypertensive disorders of pregnancy; GWG, gestational weight gain. Ref: Reference group.

Supplementary Table 2. Unadjusted and multivariate adjusted predictors of macrosomia

Characteristic	Macrosomia			
	Univariate		Multivariate	
	Unadjusted OR (95% CI)	*p*-value	Adjusted OR (95% CI)	*p*-value
Gestational age (weeks)				
<37	0.05 (0.01–0.19)	1.68E-05	0.08 (0.02–0.34)	5.13E-04
37 to <42	Ref		Ref	
≥42	5.62 (3.05–10.30)	2.87E-08	5.11 (1.92–13.60)	1.12E-03
Height	1.09 (1.07–1.10)	2.28E-39	1.07 (1.06–1.09)	4.53E-18
Pre-pregnancy BMI (kg/m^2)				
<18.5	0.50 (0.36–0.70)	3.15E-05	0.47 (0.30–0.72)	5.82E-04
18.5 to <21	Ref		Ref	
21 to <25	2.07 (1.75–2.45)	2.19E-17	2.33 (1.88–2.88)	1.21E-14
25 to <30	3.67 (2.99–4.50)	1.52E-35	4.90 (3.78–6.35)	4.62E-33
≥30	5.64 (4.32–7.35)	1.87E-37	10.00 (7.02–14.20)	2.56E-37
Total GWG (kg)				
<7	0.67 (0.51–0.87)	2.53E-03	0.36 (0.26–0.50)	1.55E-09
7 to <9	0.68 (0.53–0.88)	3.83E-03	0.69 (0.51–0.93)	1.60E-02
9 to <12	Ref		Ref	
≥12	2.29 (1.95–2.70)	7.58E-24	2.24 (1.83–2.74)	3.03E-15
Diabetes mellitus	2.34 (1.81–3.01)	5.12E-11	1.89 (1.37–2.59)	9.85E-05
History of delivery for macrosomia	14.60 (11.20–18.90)	4.40E-90	8.33 (6.32–11.00)	5.85E-51

OR, odds ratio; CI, confidence interval; BMI, body mass index; GWG, gestational weight gain. Ref=Reference group.

Supplementary Table 3. Unadjusted and multivariate adjusted predictors of moderate and severe HDP

Characteristic	Moderate HDP			
	Univariate		Multivariate	
	Unadjusted OR (95% CI)	*p*-value	Adjusted OR (95% CI)	*p*-value
History of				
HDP	9.91 (8.51–11.50)	4.73E-191	3.38 (2.79–4.09)	9.72E-36
Diabetes mellitus	2.71 (1.33–3.16)	8.64E-38	1.37 (1.13–1.66)	1.51E-03
Hypertension	42.10 (37.50–47.20)	0.00	19.90 (17.40–22.80)	0.00
Hyperthyroidism	1.63 (1.19–2.22)	2.18E-03	1.32 (0.93–1.88)	1.26E-01
Hypothyroidism	1.65 (1.21–2.24)	1.40E-03	1.50 (1.05–2.12)	2.39E-02
SLE	1.25 (0.31–5.10)	7.56E-01	0.68 (0.15–3.08)	6.18E-01
Antiphospholipid antibody syndrome	0.56 (0.14–2.26)	4.16E-01	0.48 (0.11–2.06)	3.25E-01

Characteristic	Severe HDP			
	Univariate		Multivariate	
	Unadjusted OR (95% CI)	*p*-value	Adjusted OR (95% CI)	*p*-value
History of				
HDP	14.20 (11.50–17.60)	2.28E-134	4.46 (3.44–5.79)	2.99E-29
Diabetes mellitus	2.82 (2.25–3.53)	2.15E-19	1.36 (1.02–1.82)	3.46E-02
Hypertension	39.20 (33.70–45.40)	0.00	13.20 (10.90–16.00)	8.82E-152
Hyperthyroidism	0.45 (0.19–1.08)	7.25E-02	0.32 (0.13–0.81)	1.56E-02
Hypothyroidism	2.01 (1.31–3.08)	1.32E-03	2.28 (1.43–3.63)	5.51E-02
SLE	1.49 (0.21–10.70)	6.92E-02	0.41 (0.05–3.17)	3.94E-01
Antiphospholipid antibody syndrome	1.36 (0.34–5.48)	6.68E-01	1.25 (0.28–5.51)	7.71E-01

OR, odds ratio; CI, confidence interval; HDP, hypertensive disorders of pregnancy; SLE, systemic lupus erythematosus. Ref=Reference group.

SUPPLEMENTARY FIGURE LEGENDS

Supplementary Figure 1. Flowchart for participant selection from the JECS enrollment flowchart.

BMI, body mass index.

Supplementary Figure 2. Definition of GWG in this study in various intervals during the gestational period.

The time points for measurements of pre-pregnancy height/BW and BW at first, second, and third trimesters are shown. Pre-pregnancy BMI is self-reported in the questionnaire responses. Total GWG indicates BW gain from pre-pregnancy BW to delivery. Since the timing of the visits during each trimester differed for each subject, the intervals among the specific intervals for BW gain vary among the subjects. GWG, gestational weight gain; BMI, body mass index; BW, body weight.

Supplementary Figure 3. Odds ratios for LBW and macrosomia according to GWG from pre-pregnancy to the first trimester.

The odds ratios for LBW and macrosomia in the five pre-pregnancy BMI categories according to GWG from pre-pregnancy to the first trimester are shown. GWG, gestational weight gain; LBW, low birth weight; OR, odds ratio. * $p < 0.01$ and ** $p < 0.0001$.

Supplementary Figure 4. Prevalence of GWG from the second trimester to delivery.

Percentage of GWG from the second trimester to delivery in the categories of GWG from the first to second trimesters. Four pre-pregnancy BMI categories are shown. * $p < 0.01$ and ** $p < 0.0001$.

Supplementary Figure 5. The odds ratio of HDP according to GWG during specific intervals of pregnancy.

The odds ratio of HDP in the five pre-pregnancy BMI categories according to GWG (A) from pre-pregnancy to the first trimester, (B) from the first to second trimesters, and (C) from the second trimester to delivery are shown. GWG, gestational weight gain; HDP, hypertensive

disorders of pregnancy; OR, odds ratio. * $p < 0.01$ and ** $p < 0.0001$.

Supplementary Figure 6. The odds ratio of HDP according to GWG patterns.

The upper horizontal axis shows three categories according to GWG from the first to second trimesters. The bottom horizontal axis shows four categories according to pre-pregnancy BMI. The vertical axis shows six categories according to GWG from the second trimester to delivery. The odds ratios of moderate (upper rows in cells) and severe (lower rows in cells) HDP in the 3 x 6 patterns of GWG in each pre-pregnancy BMI are shown. The cells are colored to indicate the increase in OR for moderate or severe HDP. GWG, gestational weight gain; BMI, body mass index; HDP, hypertensive disorders of pregnancy; OR, odds ratio. * $p < 0.01$ and ** $p < 0.0001$.

Supplementary Figure 1

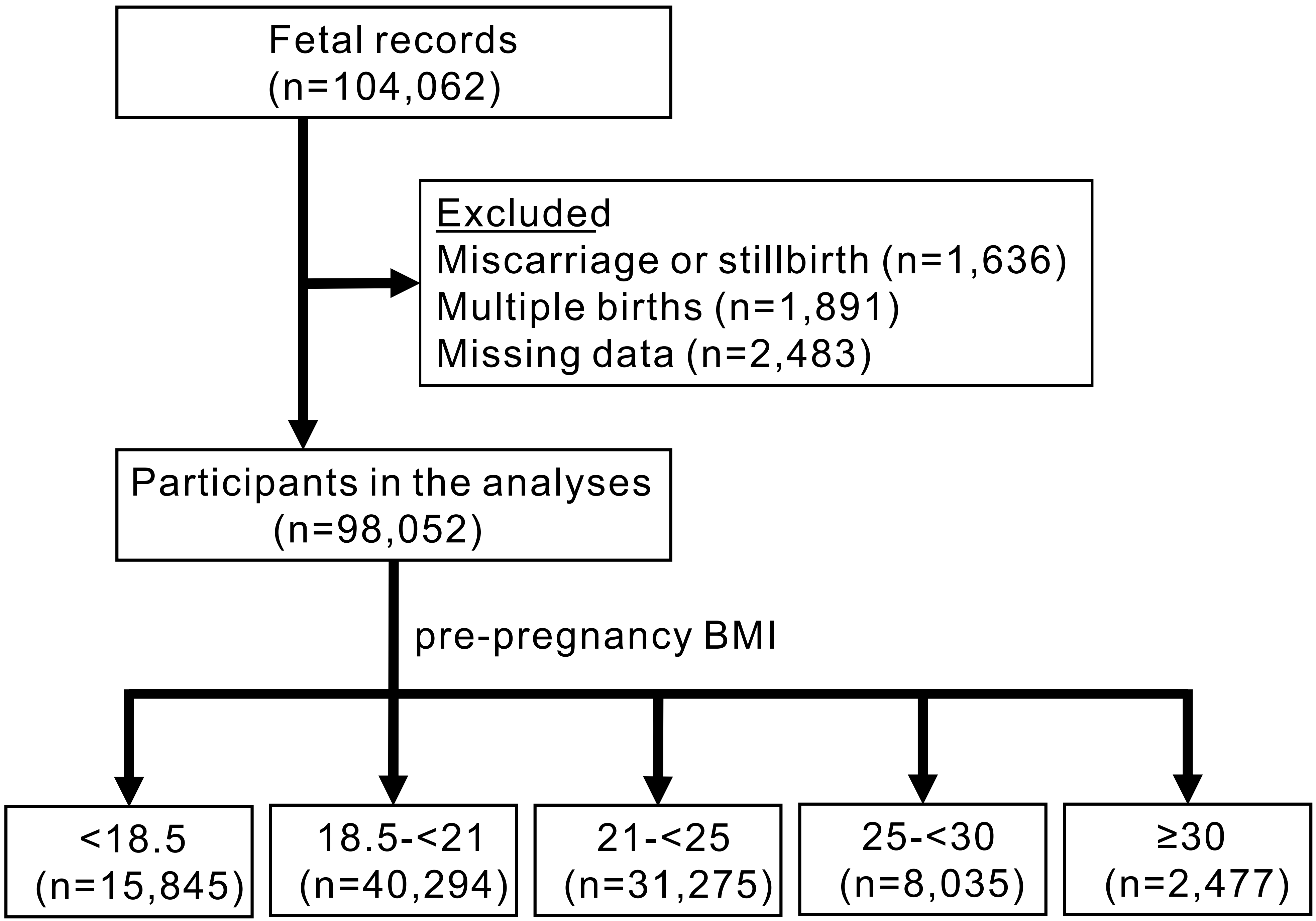

Supplementary Figure 2

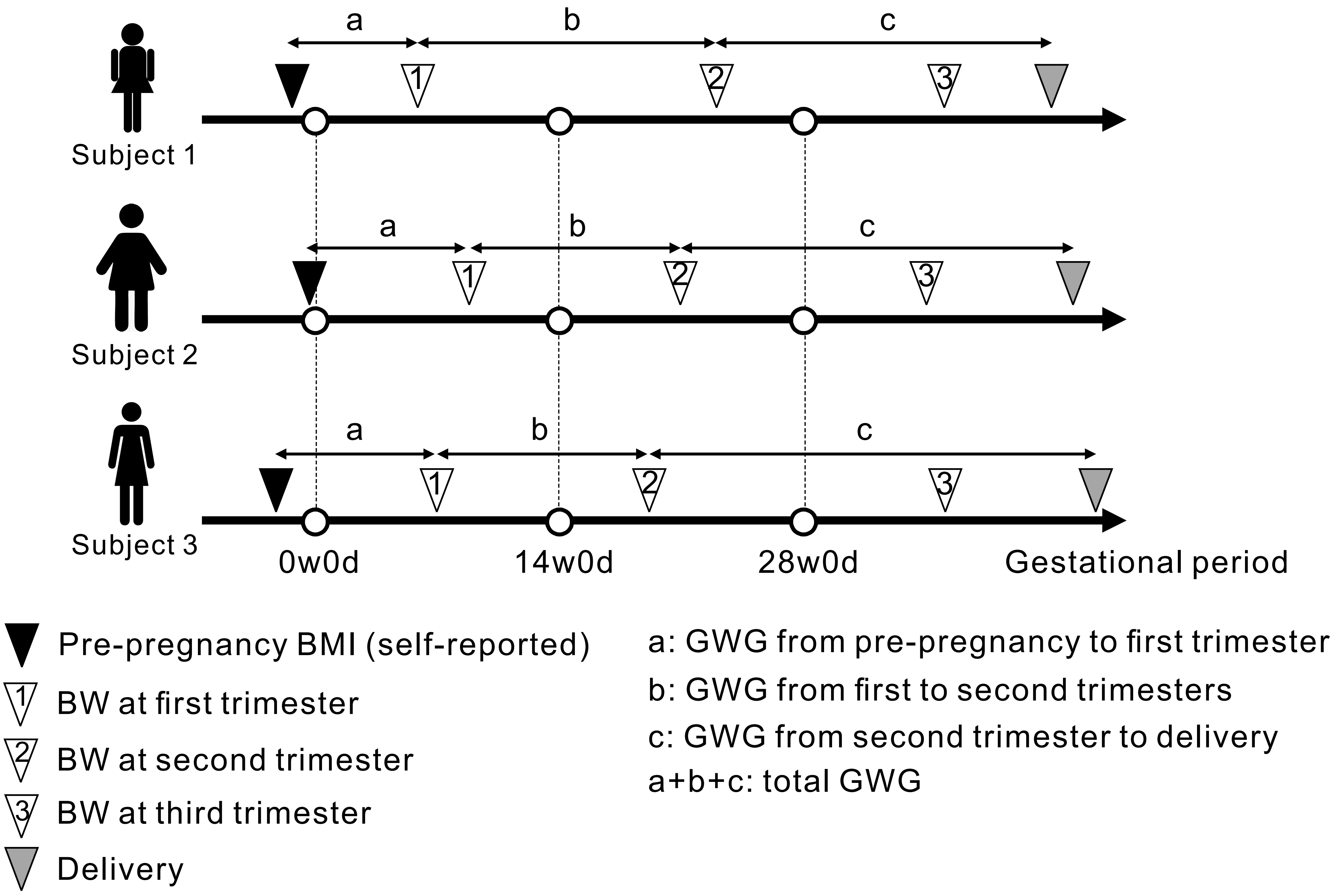

Supplementary Figure 3

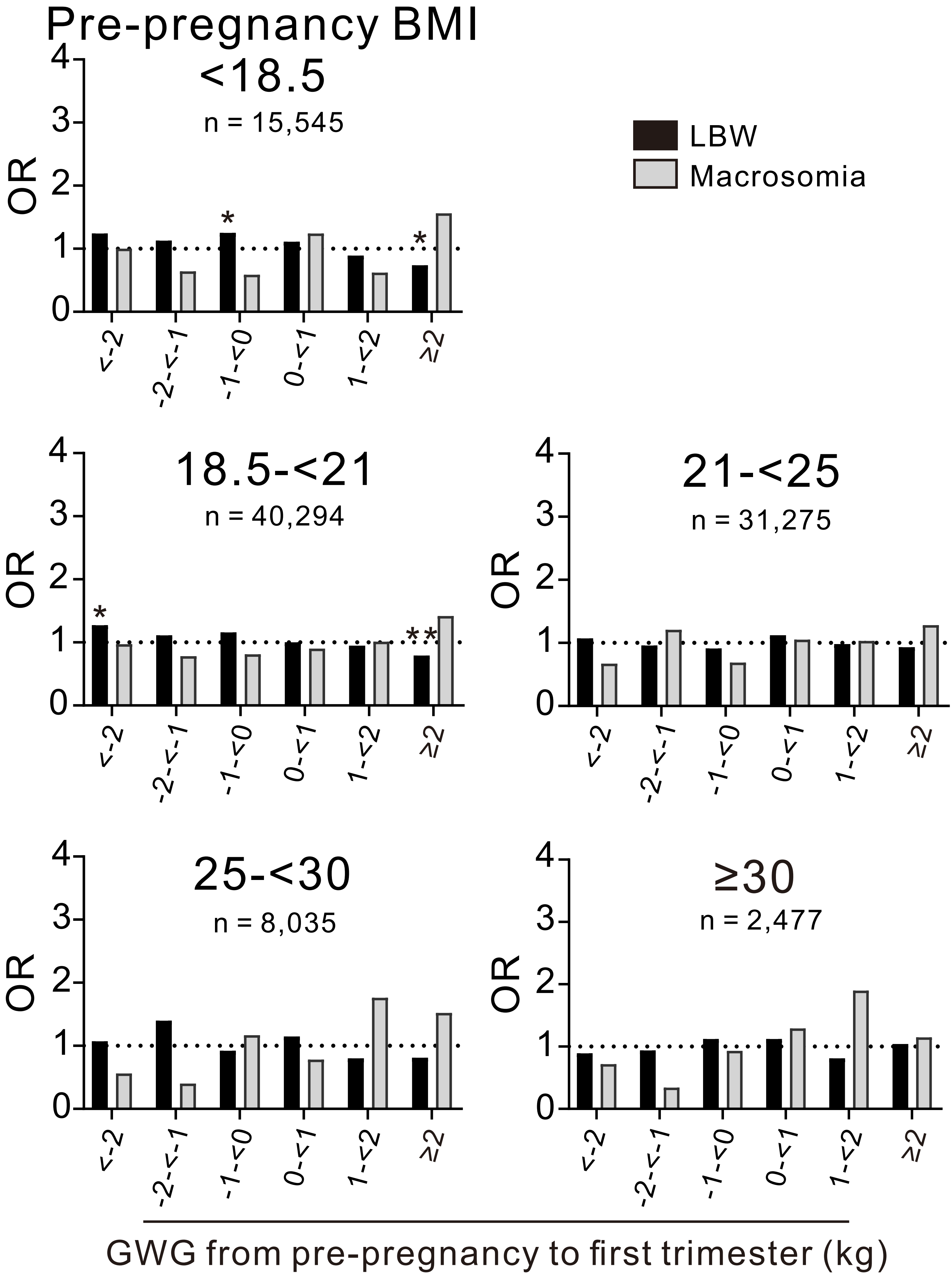

Supplementary Figure 4

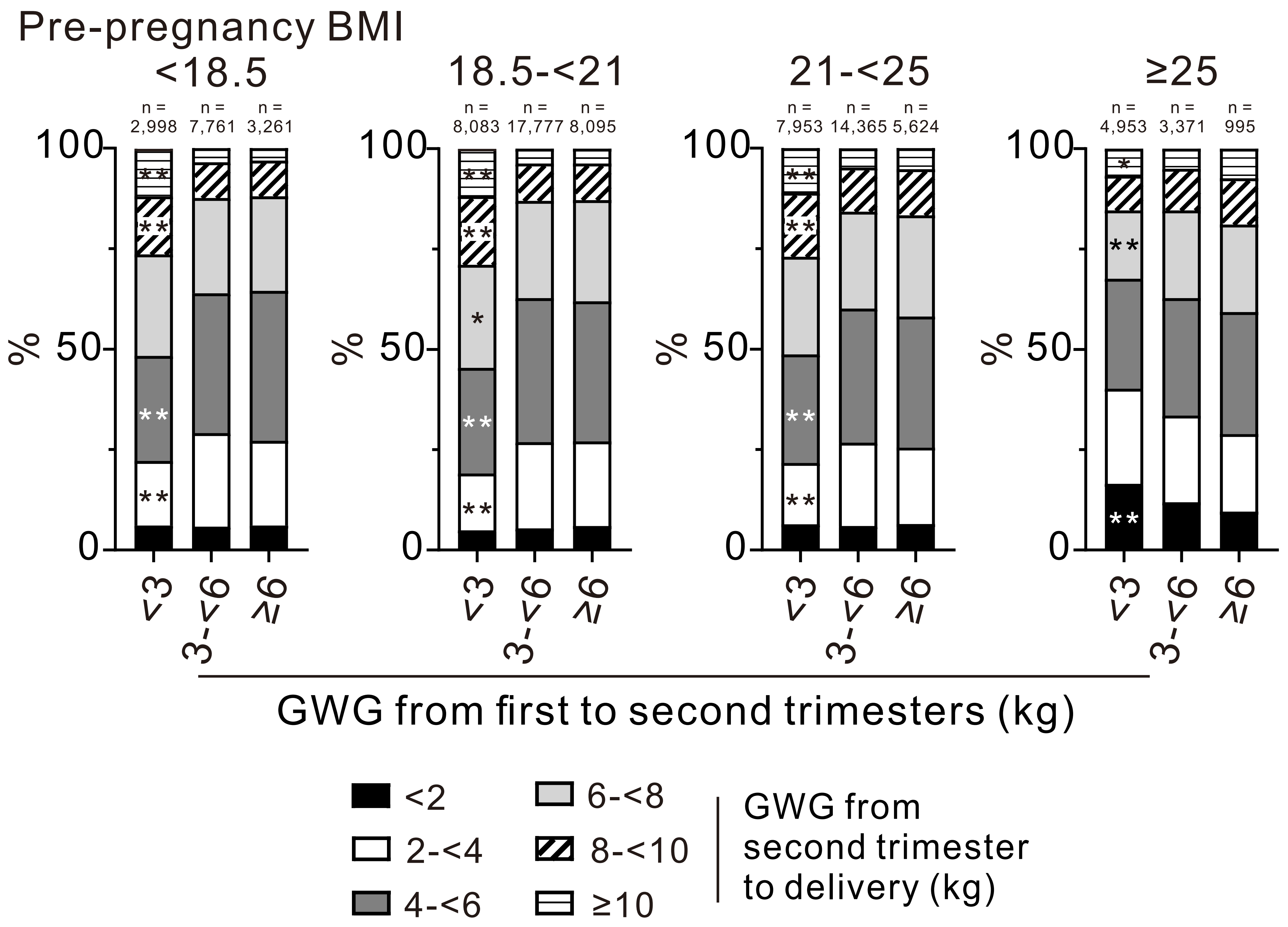

Supplementary Figure 5

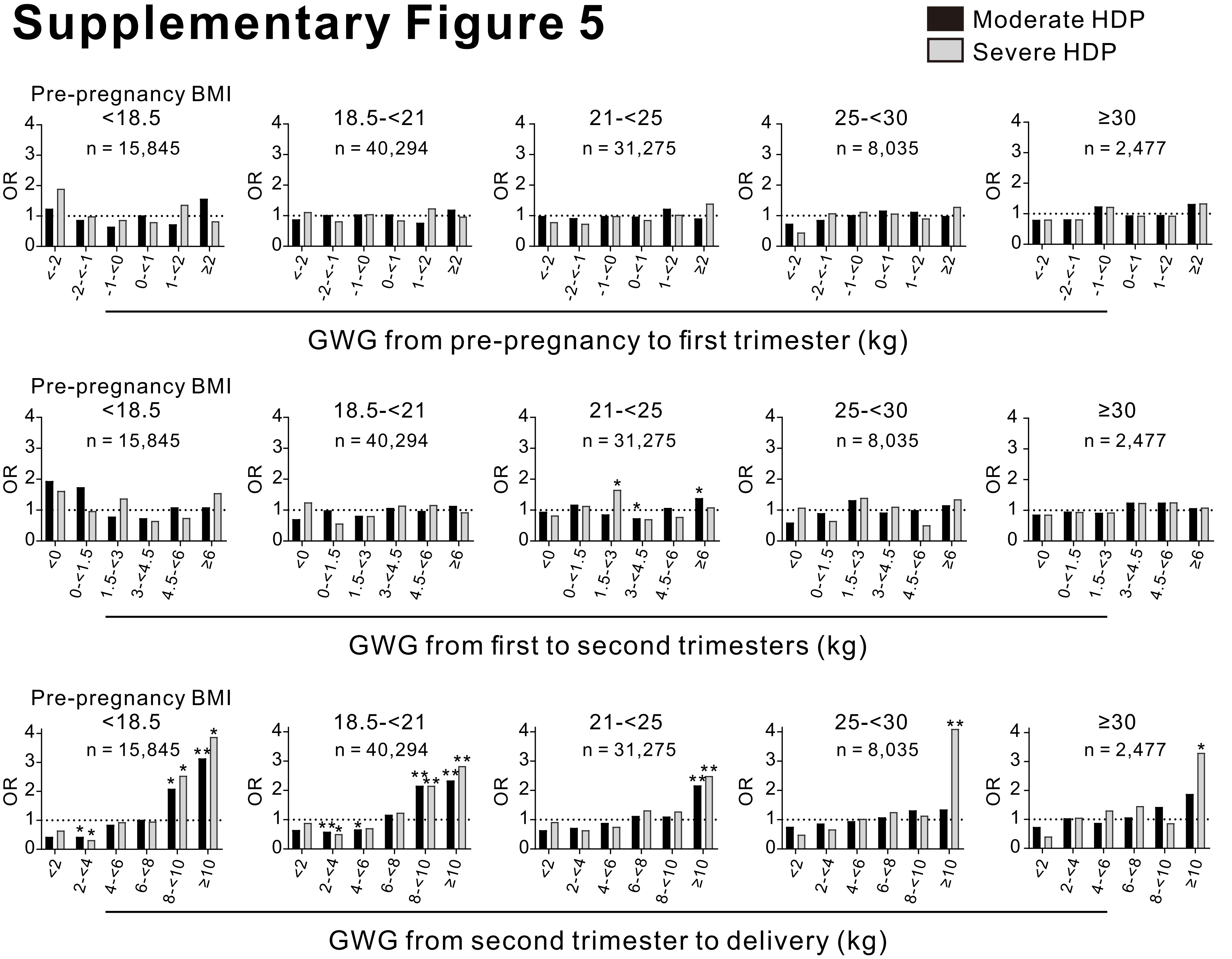

Supplementary Figure 6

OR: Moderate HDP (upper value) / Severe HDP (lower value)

- OR for moderate and severe HDP < 1.0
- OR for moderate or severe HDP 1.0-<1.5
- OR for moderate or severe HDP ≥ 1.5

Pre-pregnancy BMI	<18.5			18.5-<21			21-<25			≥25		
GWG from first to second trimesters (kg)	<3	3-<6	≥6	<3	3-<6	≥6	<3	3-<6	≥6	<3	3-<6	≥6
GWG from second trimester to delivery (kg)	n = 2,998	n = 7,761	n = 3,216	n = 8,083	n = 17,777	n = 8,095	n = 7,953	n = 14,365	n = 5,624	n = 4,953	n = 3,371	n = 955
<2 (n = 5,825)	0.40	0.76	0.18	0.70	0.49	1.09	0.45	0.67	0.84	0.80	0.62	1.19
	0.45	0.44	2.32	1.51	0.86	1.09	0.66	0.77	1.74	0.37**	0.83	0.24
2-<4 (n = 17,400)	0.49	0.45	0.43	0.55	0.56*	0.84	0.93	0.59*	0.69	0.79	1.37	1.82
	0.75	0.27	-	0.35	0.49*	0.41	0.59	0.56	0.69	0.95	0.69	0.77
4-<6 (n = 28,092)	0.84	0.98	1.04	0.67	0.68*	0.61	1.17	0.80	0.82	0.96	0.96	0.61
	1.76	1.02	0.09	0.59	0.69	0.87	0.97	0.84	0.72	1.31	1.02	0.87
6-<8 (n = 20,824)	0.92	0.78	1.35	0.61	1.42	1.10	0.97	1.24	0.89	1.12	0.82	0.78
	0.88	1.35	0.64	1.24	1.55	0.90	1.32	1.65	0.78	1.48	0.99	1.06
8-<10 (n = 9,424)	1.54	2.10	1.56	2.36*	2.23**	2.22*	0.80	1.36	1.41	1.42	1.22	1.71
	2.23	1.54	9.43**	1.11	2.98**	2.20	1.07	1.81	0.51	0.74	0.84	3.02
≥10 (n = 5,141)	2.47	4.53**	3.55	2.28*	3.14**	1.56	1.75	3.06**	2.85**	1.68	1.17	0.61
	0.51	10.5**	8.64*	2.74	2.70	5.03*	2.04	1.45	5.64*	3.31**	4.28*	1.46
